# Gelation of Poly(Vinylidene Fluoride) Solutions in Native and Organically Modified Silica Nanopores

**DOI:** 10.3390/molecules23113025

**Published:** 2018-11-20

**Authors:** Alejandra Espinosa-Dzib, Sergey Vyazovkin

**Affiliations:** Department of Chemistry, University of Alabama at Birmingham, 901 S. 14th Street, Birmingham, AL 35294, USA; maale@uab.edu

**Keywords:** calorimetry, gelation, kinetics, thermodynamics

## Abstract

The purpose of this study is to highlight the surface and size effects of the nanopores on the thermodynamics and kinetics of gelation. The effects have been probed by applying differential scanning calorimetry to poly(vinylidene fluoride) solutions in tetraethylene glycol dimethyl ether (tetraglyme) and γ-butyrolactone. Nanoconfinement has been accomplished by introducing gels into native and organically modified silica nanopores (4–30 nm). Nanoconfinement has produced two major effects. First, the heat of gelation has decreased three to four times compared to that for the bulk systems. Second, the temperature of gelation has increased by ~40 °C (tetraglyme based systems) and ~70 °C (γ-butyrolactone based systems), the increase being stronger in native nanopores. The effects are discussed in terms of acceleration of gelation due to heterogeneous nucleation at the confining surface, and retardation of gelation due to constricted polymer chain mobility in the middle of the pore volume. Calorimetric data have been subjected to isoconversional kinetics analysis. The obtained temperature dependencies of the activation energies of gelation have been interpreted in the frameworks of the nucleation model of Turnbull and Fisher. The results suggest that nanoconfinement leads to a lowering of both the free energy of nucleation and activation energy of diffusion.

## 1. Introduction

Many polymer solutions can gel. A gel is a special state when the solution loses its ability to flow, i.e., turns into the so-called soft solid. Gels are formed at the expense of crosslinking of polymer chains that form a three-dimensional network that entraps large amounts of a solvent. Nanogels are soft materials, whose size is less than 0.5 μm [1]. Because of their small size, nanogels find promising applications in nanomedicine as drug-delivery, imaging, and therapeutic materials [2]. High loading capacity and stability, responsiveness to the ionic strength, pH and temperature, biocompatibility, and biodegradability are among the gel properties that are hard to find in other types of colloidal systems [3]. Nanogels can circulate inside the body, entering the cells for burst release to deliver drugs and target diseases more effectively [3,4,5]. Photolithographic, micromolding, microfluidic techniques as well as free radical and emulsion polymerization are several methods for the fabrication of nanogels [4]. At the same time, nanogels can be prepared by introducing polymer solutions in nanopores of solid materials, such as silica gel [6].

Combining nanogels with nanosized solids, such as quantum dots, nanoparticles, and nanofibers, leads to the enhancement of nanogel properties and expansion of their application area [2,7,8]. In particular, porous silica provides a well-ordered mesostructured template [9], the surface silanol groups of which can be flexibly modified for a variety of nanotechnology applications [10,11]. The surface silanol groups are capable of hydrogen bonding as well as of ionizing, which may lead to strong ion-dipole and ion-ion interactions [12,13]. On the other hand, organic modification replaces silanol groups with hydrocarbon moieties that give rise to a hydrophobic surface capable of only weak van der Waals interactions [14,15].

Confining a polymer solution to nanopores should be expected to affect its ability to gel in general and its gelation kinetics in particular. First, confining a polymer chain restricts its mobility, especially when the size of confining space is comparable or smaller than that of the gyration radius, R_g_. In this circumstance, one may expect gelation to decelerate relative to the unconfined process, taking place in the bulk solution. Second, the presence of a nanoconfining surface may accelerate gelation. This is easy to understand considering that for numerous polymer solutions, the creation of the crosslinks in physical (thermoreversible) gels has been associated with the formation of microcrystallites [16,17]. Then, the presence of a confining surface may be expected to induce the formation of the microcrystallites via heterogeneous nucleation that faces smaller energy barriers, and thus occurs faster than homogenous nucleation in the bulk solution. It means that acceleration of gelation is another possible effect. In turn, the extent of acceleration can be expected to differ depending on the nature of the confining surface. As mentioned earlier, the native silica surface is capable of stronger interactions than the organically modified one. That is, gelation of a polymer, which is polar and/or capable of hydrogen bonding, should be affected more strongly by the native silica surface and more weakly by the organically modified silica surface. Therefore, we can hypothesize that, under nanoconfinement, the ability of a polymer solution to gel should be influenced by the size of the silica pores as well as by the nature of the silica surface. Needless to say, the aforementioned effects should manifest themselves via basic thermodynamic and kinetic parameters, as already demonstrated in a study of solid-solid transitions confined to native and organically modified nanopores [18].

Although nanogels are intensively studied, the physics of nanoconfined gelation in polymer solutions is largely unexplored. There have been a few interesting reports relevant to gelation under confinement in general, i.e., not necessarily under confinement to nanosizes. One such report is on a study of an aqueous solution of polyethylene oxide [19] (PEO), with the inclusion of polystyrene spheres of different diameters, 0.78, 1, and 1.55 μm, placed between two glass plates. Microrheological analysis of the solution has demonstrated that when the scale of nanoconfinement approaches the magnitude of R_g_, the solution gels. This is a remarkable result because the aqueous solutions of PEO are not known to gel in the bulk state. Apparently, confinement can promote gelation. A similar trend has been revealed for the sol-gel transition of the solution of 1,3:2,4-Di-p-methylbenzylidene sorbitol in propylene carbonate that has been studied in lithographically constructed microchannels of a 20–80 µm size [20]. Differential scanning calorimetry (DSC) measurements have revealed that on cooling, the confined solution gels at higher temperature than the bulk one. That is, confinement again has promoted gelation. Gelation of *N*-lauroyl-*L*-glutamic acid di-*n*-butyamide solutions in ethylene glycol and propylene glycol has been studied between two parallel cover glasses [21,22]. The gelation kinetics in propylene glycol has been monitored as the evolution of the elastic modulus and found to slow down significantly as the gap between the cover glasses dropped below 100 μm [22]. Therefore, confinement has suppressed gelation in this case. 

In all aforementioned cases, confinement has occurred on the micrometer rather than nanometer scale. Nanoconfinement has been accomplished by introducing 1-methyl-2,4-bis(*N*′-n-octadecylureido)benzene, bis(4′-stearamidophenyl)methane, and bis(4′-stearamidophenyl)methane solutions in chlorobenzene and propylene carbonate into 2–3 nm size galleries of organically modified montmorillonite clay [23]. The study has reported that nanconfinement enhances the thermal stability of the gels as detected by increasing the melting temperature measured by DSC. A similar conclusion has been arrived at in a study of an aqueous solution of gelatin confined to nanopores of native silica [6]. Neither of these two publications, however, has been focused on the process of gelation. The rate of gelation has been measured in two polystyrene/clay nanocomposites dissolved in carbon disulfide [24]. In one system, the polystyrene chains have been partially intercalated inside the clay gallery, while in another, they have been tethered to the clay surface to form a brush structure. In both systems, the polymer chains experience nanoconfinement in the vicinity of the clay surface [25]. The gelation rate for the polystyrene brush solution has been practically identical to that for the bulk solution. However, the gelation rate of the intercalated polystyrene has increased significantly, which has been explained by possible heterogeneous nucleation [24].

As follows from the above there have been no systematic studies of the effects of the size of nanoconfinement and the nature of the nanoconfining surface on the thermodynamics and kinetics of the gelation process. Nevertheless, such studies are of fundamental importance for the rational development of nanogel materials. The major objective of the present work is to initiate the aforementioned systematic studies. As discussed above, insights into the effect of the nature of the nanoconfining surface can be accomplished via comparative examination of gelation in native and organically modified silica nanopores. The need of employing the latter immediately eliminates the possibility of using the most common type of polymer gels, hydrogels, because aqueous solutions cannot penetrate the hydrophobic nanopores. Therefore, a model system has to be a polymer solution in an organic solvent. Another requirement for choosing the model system arises from our previous experience of successfully studying gels by means of calorimetry [6,24,26,27,28,29,30,31]. This requirement is for gelation to occur with a readily detectable heat release. With the account of the above requirements, our choice has fallen on polyvinylidene fluoride (PVDF), which is known to undergo gelation with a variety of organic solvents as well as with a significant change in the enthalpy [32,33]. In particular, for the PVDF-γ-butyrolactone (BL) and PVDF-tetraglyme (TG) gels chosen for the present studies, the respective enthalpy changes have been reported as 13 and 10 J g^−1^ [32].

In order to accomplish the stated objective, the present work focuses on calorimetric studies of gelation of PVDF-TG and PVDF-BL solutions in the bulk state as well as in the state confined to native (Nat) and organically modified (OM) silica nanopores of different sizes. The intention is to employ calorimetry for obtaining basic thermodynamic and kinetic information about respective gelation processes. The obtained information is then used for obtaining insights into the effects of the size of nanoconfinement and the nature of the nanoconfining surface on the process of nanoconfined gelation. For brevity, the systems under study are denoted as “PVDF/Solvent (i.e., TG or BL)/Pores type (i.e., Nat or OM)”. For example, the bulk TG based system is denoted as “PVDF/TG”, whereas the same system confined to OM nanopores as “PVDF/TG/OM”.

## 2. Results and Discussion

### 2.1. FTIR and XRD Characterization

As stated above, the gel crosslinks are microcrystallites. FTIR and XRD analyses have been conducted in order to try to establish the crystalline form of the crystallites and see if it remains unchanged between the bulk and nanoconfined gels. This is a very challenging task, especially in the case of nanoconfined gels when the instrumental signal is complicated by the presence of silica and is very weak due to a small extent of “crystallinity”. The latter can be estimated by comparing the heats of gelation reported further and the heat of melting of 100% crystalline PVDF, which is 6.70 kJ mol^−1^ [34], or 105 J g^−1^. Based on this, the “crystallinity” of the nanoconfined PVDF/TG and PVDF/BL gels is ~9% and ~6%, respectively.

**Figure 1:**
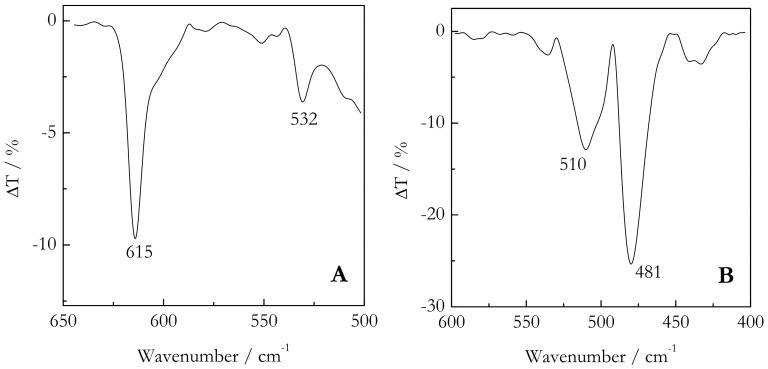
FTIR spectra of the bulk PVDF/TG (**A**) and PVDF/BL (**B**) gels. The vertical axis represents the difference in the transmittance between the gel and solvent.

PVDF may crystallize in three major forms denoted as α, β, and γ (also known as forms I, II, and III), which have specific infrared absorption bands and X-ray reflections peaks. Important conformation specific infrared absorptions of PVDF are found below 650 cm^−1^. The FTIR spectra for the bulk systems PVDF/TG and PVDF/BL are shown in Figure 1A,B. The PVDF/TG gel clearly shows (Figure 1A) absorption bands at 532 and 615 cm^−1^. Both are specific of the α phase [35,36]. Figure 1B presents FTIR spectra for the bulk PVDF/BL gel. There are two strong peaks that appear in 481 and 510 cm^−1^, which are characteristic of the γ-phase [37,38]. FTIR spectra of nanoconfined gels have been dominated by strong absorption bands of silica that made it impossible to identify any specific phases of PVDF.

**Figure 2:**
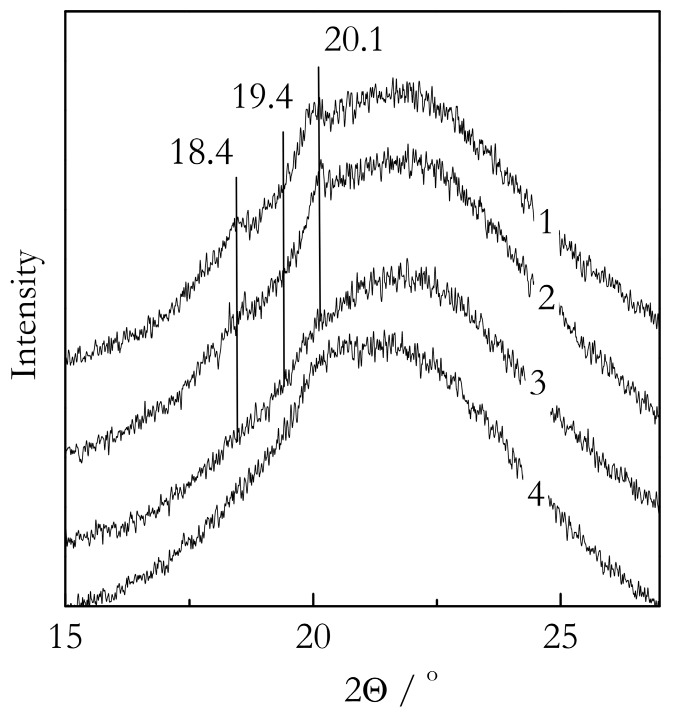
X-ray difractograms of the PVDF nanoconfined gels in 30 nm silica pores: PVDF/TG/NAT (1), PVDF/TG/OM (2) PVDF/BL/NAT (3), and PVDF/BL/OM (4).

X-ray diffraction patterns for gels confined to native and organically modified silica are displayed in Figure 2, which depicts XRD data for nanoconfined systems. Our analysis follows theoretical calculations by Jurczuk et al. [37], who have reported calculated values of the diffraction angles (2θ) for all phases of PVDF and validated them against experimentally observed values. According to their report, in the region of smaller angles, the α and γ phases produce XRD peaks at 18.4° and 20.1°. In addition, the γ phase also produces a peak at 19.4°, which is not observed in the α phase. Therefore, this peak can be used to identify the γ-phase. For both PVDF/TG/Nat and PVDF/TG/OM, we observe small diffraction peaks around 18.4° and 20.1°, but no peaks around 19.4°. Thus, we can conclude that the crystallites in the nanoconfined TG-based gels retain the α-phase as the bulk gels. As expected, analysis of the nanoconfined BL-based gels is more challenging. Nevertheless, the PVDF/BL/Nat system shows a small peak around 20.1°. The peak is markedly smaller than that in the TG systems because of the significantly lower “crystallinity”. More importantly, the PVDF/BL/Nat system shows some weak diffraction around 19.4°. While weak, the signal rises above the detection limit and thus provides some evidence that the crystallites formed under nanoconfinement retain the γ-phase as detected for the bulk. Unfortunately, the PVDF/BL/OM system has not produced any distinct diffraction peaks that would rise above the detection limit. However, considering that nanoconfinement of the TG based systems has not introduced any phase change and that nanoconfinement of the BL system to Nat pores has not introduced it either, it does not seem unreasonable to expect the PVDF/BL/OM system also retains the same phase as its respective bulk. 

### 2.2. DSC Data on Gelation of the Bulk Systems

**Figure 3:**
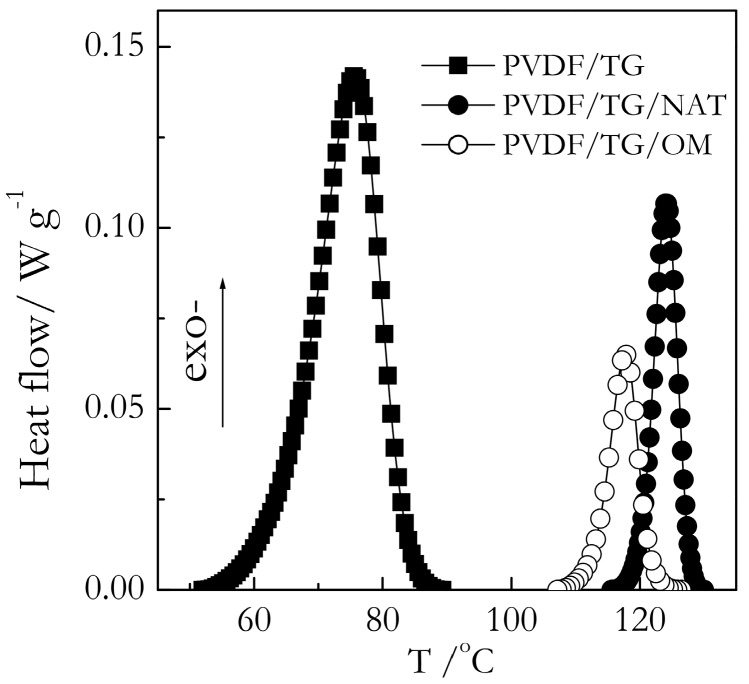
DSC curves for TG-based gels at a cooling rate of 8 °C min^−1^. The bulk gel is represented by squares, PVDF/TG/NAT and PVDF/TG/OM gels confined in 9 nm pores represented by solid and open circles, respectively. The heat flow values are given per gram of gel.

**Figure 4:**
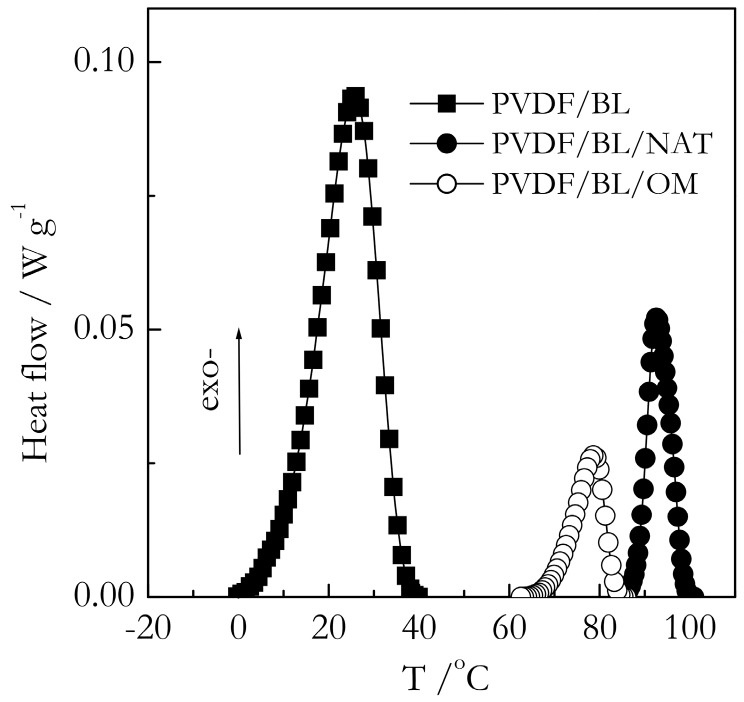
DSC curves for BL-based gels at a cooling rate of 8 °C min^−1^. The bulk gel is represented by squares, PVDF/BL/NAT and PVDF/BL/OM gels confined in 9 nm pores represented by solid and open circles, respectively. The heat flow values are given per gram of gel.

Some representative DSC data on gelation of the bulk and nanoconfined systems are presented in Figure 3 and Figure 4. Both TG and BL systems show two similar effects. First, we can see that for the nanoconfined systems, DSC peaks appear at higher temperatures than for the bulk. Because the process is measured on cooling, the appearance of a peak at a higher temperature means that the process occurs sooner, i.e., it is faster. Second, the DSC peak areas for the nanoconfined systems are noticeably smaller, which means that gelation occurs to a smaller extent than in bulk.

**Figure 5:**
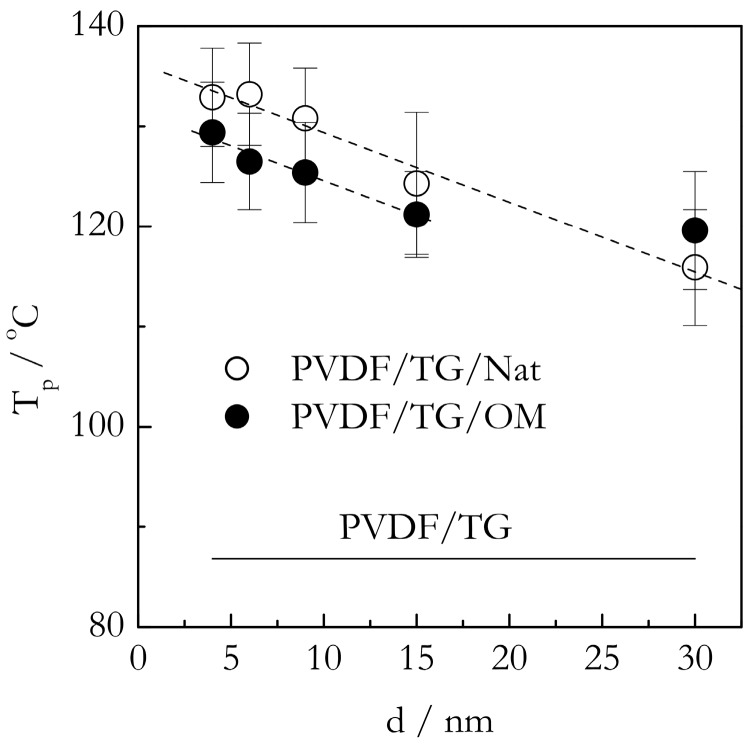
DSC peak temperatures for gelation of PVDF/TG bulk (solid line), and confined systems, PVDF/TG/Nat (open circles), PVDF/TG/OM (solid circles) vs. the pore diameter. Dashed line is a guide to the eye.

**Figure 6:**
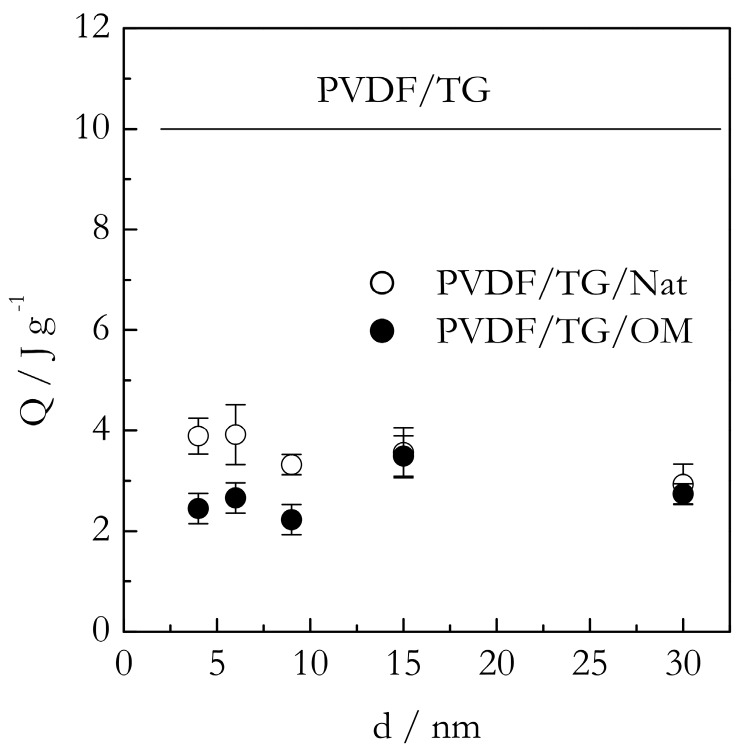
Heat of gelation of PVDF/TG in bulk (solid line) and confined systems PVDF/TG/Nat (open circles), PVDF/TG/OM (solid circles) dependence on the pore diameter size. The values of Q are given per gram of gel and represent the average of all cooling rates.

**Figure 7:**
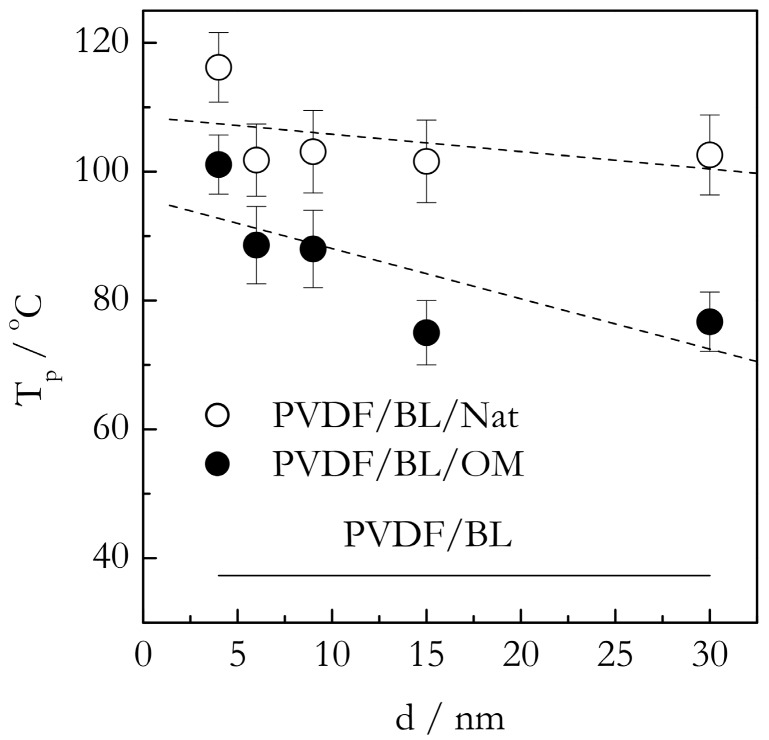
DSC peak temperatures for gelation of PVDF/BL in bulk (solid line) and confined systems PVDF/BL/Nat (open circles), PVDF/BL/OM (solid circles) vs. the pore diameter. Dashed line is a guide to the eye.

**Figure 8:**
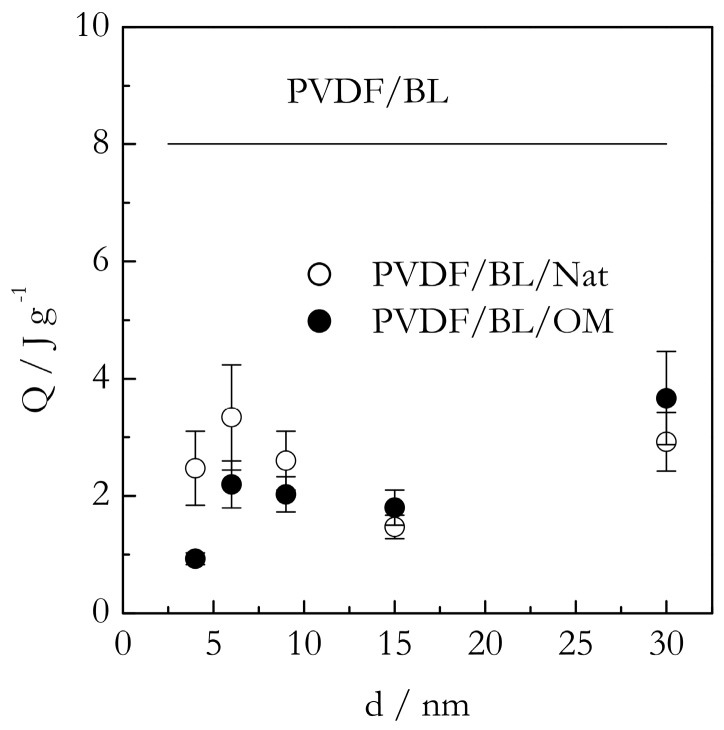
Heat of gelation of PVDF/BL in bulk (solid line) and confined systems PVDF/BL/Nat (open circles), PVDF/BL/OM (solid circles) dependence on the pore diameter size. The values of Q are given per gram of gel and represent the average of all cooling rates.

Figure 5 shows the DSC peak temperatures (T_p_) for the gelation of the PVDF/TG system in the bulk (solid line) and nanoconfined state (points). The T_p_ values have been estimated as the mean value of the peak temperatures obtained at five cooling rates from the interval, 0.5–8 °C min^−1^. For the bulk system, this value is ~86 °C. The heats of gelation (Q) have been estimated in a similar manner to yield the value of ~10 J g^−1^ (Figure 6). Here, and elsewhere, the heats are reported per gram of gel. These results are in reasonable agreement with Voice et al. [39], who reported that gelation of the 30% wt. PVDF/TG system occurs around 76 °C at the cooling rate of 2 °C min^−1^ and the heat release is about 11 J g^−1^. The DSC peak temperatures for the gelation of the bulk (solid line) and nanoconfined (points) PVDF/BL systems are presented in Figure 7. The mean temperature of gelation for the bulk system is 36 °C and the mean heat of gelation is ~8 J g^−1^ (Figure 8). 

A comparison of the bulk systems indicates that there is a significant effect of the solvent type on the parameters of gelation. This is an important fact that means that the solvent participates in the formation of the gel junctions [17]. The significant difference in the gelation temperature of the bulk systems can be linked to the power of the respective solvents. BL is a much stronger solvent than TG. Its dipole moment is 4.2–4.3 D [38,40,41]. The polarity of TG is only 2.4–2.5 D [42,43]. Thus, one should expect a stronger solvent-polymer interaction in the BL solution. On cooling the solvent, the power decreases, and the solvent-polymer interactions weaken, which allows the chains to aggregate and form the gel junctions. Naturally, the stronger a solvent, the more the temperature needs to be lowered to form a gel.

### 2.3. DSC Data on Gelation of Nanoconfined Systems

Figure 5 presents the most important effect discovered in the present study. This is a dramatic increase in the gelation temperature of nanoconfined systems relative to the bulk one. As seen in Figure 5, in nanoconfined systems, gelation occurs roughly at 40 °C higher than in the bulk. The occurrence of gelation at higher temperatures means that the process requires less cooling, i.e., occurs easier than in the bulk system. This result is obviously consistent with our hypothesis of the acceleration due to initiation of heterogeneous nucleation in the presence of the nanoconfining surface. A closer look at Figure 5 reveals two other effects that support the same idea. First, the gelation temperature appears to increase with decreasing the pore size. This trend is not particularly strong. In addition, one of the systems (PVDF/TG/OM in 30 nm pores) clearly falls out, which, considering the size of the error bars, can be due to a statistical fluctuation. However, statistical analysis of the correlation between T_p_ and the pore diameter, d, for both OM and Nat pores yields the values of the Spearman’s coefficient of rank correlation [44] that are statistically significant at a 95% level of confidence. It means that the trend is rather unlikely to be accidental. The trend is obviously consistent with the idea of heterogeneous nucleation because a smaller pore diameter means a larger surface area and, therefore, more intense nucleation at the surface. The fact that the trend is relatively weak suggests that the effect might be close to saturation. That is, 30 nm pores provide enough surface area interaction to cause nearly maximum possible acceleration. In this situation, a further increase in the surface area cannot cause much further acceleration. The second effect is that the gelation temperatures for the PVDF/TG system confined in the Nat pores are, on average, about 5 °C larger than those for the system confined in OM pores. Once more, this is in agreement with our hypothesis that heterogeneous nucleation should manifest itself stronger in the case of the native surface that is capable of stronger interaction with PVDF.

The system PVDF/BL demonstrates an even larger increase in the gelation temperature under nanoconfinement (Figure 7). Again, the system nanoconfined in the native pores shows an increase of roughly 70 °C. The increase is noticeably larger than the corresponding increase in the PVDF/TG/Nat system, which is about 40 °C. The stronger effect observed in the PVDF/BL/Nat system is likely due to the fact that BL is much more polar solvent than TG (4.2 vs. 2.5 D vide supra). As noted earlier, the dependence of the gelation temperature on the type of solvent indicates that the solvent is involved in the formation of the gel network junctions. It is, therefore, natural to expect that the PVDF chains solvated with BL would interact more strongly with the native surface than those solvated with TG. Thus, gelation of the BL based system in the presence of the native surface should experience stronger heterogeneous nucleation and, thus, larger acceleration relative to the bulk system. This ultimately causes a larger increase in T_p_ for the PVDF/BL/Nat system relative to PVDF/BL than for the PVDF/TG/Nat system relative to PVDF/TG.

Apparently, the large polarity of BL is also the reason why the difference in the gelation temperature between the PVDF/BL/Nat and PVDF/BL/OM systems is noticeably larger than the respective difference between the PVDF/TG/Nat and PVDF/TG/OM systems: The former is about 15–20 °C whereas the latter is about 5 °C. If we look at the increases in T_p_ for the PVDF/TG/OM system relative to the PVDF/TG bulk and for the PVDF/BL/OM system relative to the PVDF/BL bulk, they seem rather comparable: Roughly 40 and 50 °C. This is because the major type of interaction between the PVDF chains solvated with TG and/or BL and the nonpolar OM surface is via van der Waals forces. In this situation, the strongly polar BL can add only little via dipole-dipole interactions with the surface methyl groups (the dipole moment of a C-H group is 0.4 D [14]). However, this type of interactions can add significantly to the interaction with native surface (the dipole moment of a O-H is 1.5 D [14]). Note that the energy of the interaction of dipoles increases in proportion to the product of the dipole moments [14]. If, for two 1 D dipoles, it is about 2 kJ mol^−1^, for 1.5 D (O-H) and 4.2 D (BL), it would be more than 12 kJ mol^−1^, i.e., comparable in energy to hydrogen bonding. This can explain why the gelation temperature in the PVDF/BL/Nat system is about 15–20 °C higher than in the PVDF/BL/OM system. On the other hand, the gelation temperature in the PVDF/TG/Nat system is only about 5 °C higher than in the PVDF/TG/OM system because TG is not polar enough to induce any significant polar-polar interactions with either the OM or native surface. 

The effect of the pore diameter on the gelation temperature in the nanoconfined PVDF/BL systems is not as straightforward as in the case of the TG based systems. Analysis of the Spearman’s coefficient of rank correlation suggests that there is a statistically significant trend of increasing T_p_ with decreasing the pore size for the PVDF/BL/OM system. However, no statistically significant trend has been detected for the PVDF/BL/Nat system. As already discussed, the existence of a weak trend or the absence thereof is a likely sign of the effect being close to saturation. Overall, the gelation temperature behavior of the BL based systems is generally quite similar to that observed for TG based systems. For both systems, it is consistent with our hypothesis of the nanoconfining surface acting as a promoter of heterogeneous nucleation and, therefore, of the accelerated gelation that occurs at markedly higher temperatures relative to gelation in the bulk systems. 

Comparison of the gelation heats of the nanoconfined and bulk systems (Figure 6 and Figure 8) brings about further important insights. Since the effects observed for the TG and BL based systems are very similar they are discussed simultaneously. Relative to the bulk value, either system demonstrates a significant drop in the heat of gelation. Roughly, the average heat of gelation for the nanoconfined TG systems is about 3 J g^−1^, and for the nanoconfined BL systems, it is around 2 J g^−1^. This constitutes a three to four times decrease relative to the heat of gelation in the respective bulk systems. It is important to recognize that the heat of gelation is proportional to the number of crosslinks formed in the gel. In other words, under the nanoconfined conditions, we obtained gels containing significantly fewer crosslinks. This may seem in contradiction with the afore-discussed effect of gelation being promoted (accelerated) in the presence of the nanoconfining surface. This is only a seeming contradiction. As follows from the above discussion, gelation is promoted in vicinity of the surface. It does not mean that it is also promoted in the middle of the pore volume. On the contrary, the polymer chain mobility is highly restricted and hampers the process of crosslinking, which is another effect, the existence of which has been hypothesized in the introduction. 

Therefore, under nanoconfinement, we have a combination of two effects. The surface effect accelerates gelation in the vicinity of the surface and causes the process to occur at higher temperatures. The size effect retards gelation in the middle of the pore volume and causes the process to create fewer crosslinks and thus to occur with a smaller heat release. The effects are obviously in competition with each other, which provides an explanation to other noteworthy experimental results presented in Figure 6 and Figure 8. This is the absence of the size effect on the heat of gelation. One may naturally expect that decreasing the pore size should increase the surface area, and, therefore, the heat of gelation. However, this effect is counteracted by the fact that as the pore size decreases, the polymer chain mobility decreases as well, leading to the formation of fewer crosslinks and, thus, to the smaller heats of gelation. Overall, decreasing the pore size yields more crosslinks near the surface and fewer crosslinks throughout the volume. The former effect increases the heat of gelation while the latter decreases it so that they practically cancel each other, resulting in the total heat of gelation being independent of the pores size. 

### 2.4. Kinetics of Gelation

The occurrence of gelation in the nanopores at significantly higher temperatures than in the bulk has prompted us to propose that the effect is associated with heterogeneous nucleation promoted by the confining surface. To further test this idea, we have conducted kinetic analysis of gelation. As already stated, the creation of the gel network junctions is commonly accepted to occur via the formation of microcrystallites [16,17]. This is the reason that the kinetics of physical gelation is phenomenologically similar [45] to the kinetics of crystallization [46,47,48]. This justifies the use of the classical nucleation model by Turnbull and Fisher [49] for describing the kinetics of gelation [27,50]. According to this model the rate of nucleation depends on temperature as follows:

(1) $$\;w\left(T\right)=w_{0}\exp\left(\frac{-\Delta G^{*}}{RT}\right)\exp\left(\frac{-E_{D}}{RT}\right)\;$$

where ΔG* is the free energy of nucleation, *E_D_* is the activation energy of diffusion, *w*_0_ is the preexponential factor, *R* is the gas constant, and *T* is the absolute temperature. In principle, this equation can be used to evaluate the effect of nanoconfinement on the kinetics of a nucleation driven process. It has been employed to evaluate such an effect on the kinetics of the morphological solid-solid transition [18,51], and coil-to-globule transition [52]. This present study appears to be the first attempt using the Turnbull-Fisher model for parameterizing the effect of nanoconfinement on the process of gelation. Following the previous work [18,27,51,52], we will combine Equation (1) with the isoconversional principle [53] that yields an equation for a temperature dependence of the isoconversional activation energy:

(2) $$\;E_{\alpha }=E_{D}-A\left[\frac{2T}{{\left(\Delta T\right)}^{3}}-\frac{1}{{\left(\Delta T\right)}^{2}}\right]\;$$

where *A* is a constant that includes the constituent parameters of ΔG* that are independent of temperature, and Δ*T* = *T_m_* − *T* is the supercooling relative to the melting temperature, *T_m_*. The parameters of Equation (2) can be obtained by fitting it to the experimental temperature dependence of *E_α_* derived by means of an advanced isoconversional method [54]. It evaluates *E_α_* by minimizing the function:

(3) $$\;\Psi \left(E_{\alpha }\right)=\sum_{i=1}^{n}\sum_{j\neq i}^{n}\frac{J\left[E_{\alpha },T_{i}\left(t_{\alpha }\right)\right]}{J\left[E_{\alpha },T_{j}\left(t_{\alpha }\right)\right]}\;$$

where:

(4) $$\;J\left[E_{\alpha },T_{i}\left(t_{\alpha }\right)\right]\equiv \int_{t_{\alpha -\Delta \alpha }}^{t_{\alpha }}\exp\left[\frac{-E_{\alpha }}{RT_{i}\left(t\right)}\right]\;$$

The method makes use of the data obtained at *n* different temperature programs, *T_i_*(*t*), e.g., different cooling rates. Integration is carried numerically over time segments corresponding to small intervals of conversion, Δ*α*, within which *E_α_* is assumed constant. This permits the elimination of a systematic error in evaluating *E_α_* when it varies significantly throughout the whole interval of conversions. The values of conversion are determined directly from DSC data as the partial peak area.

**Figure 9:**
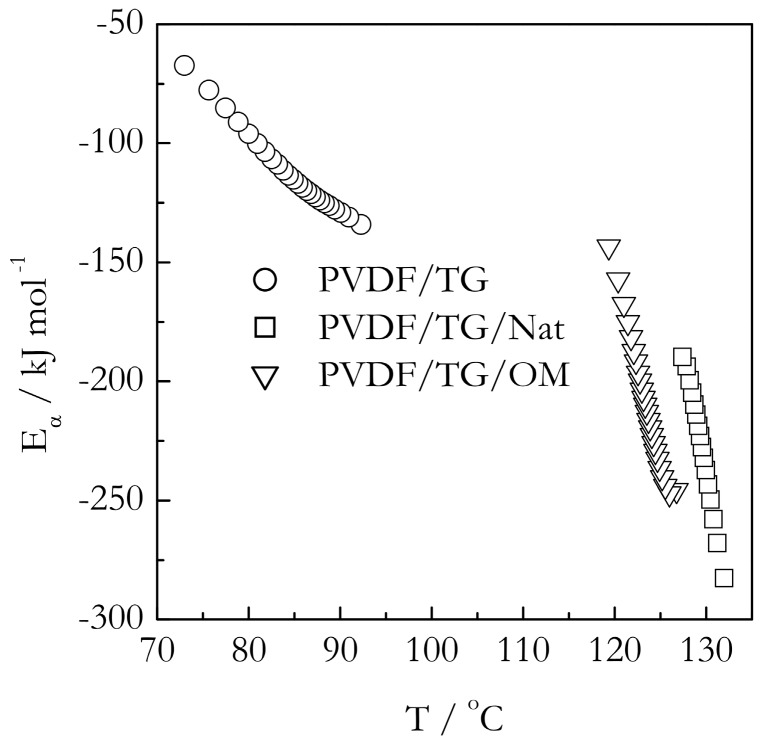
Temperature dependence of activation energy for gelation of PVDF/TG in bulk (circles) and confined systems PVDF/TG/Nat (triangles) and PVDF/TG/OM (squares). Confinement is to 9 nm pores.

**Figure 10:**
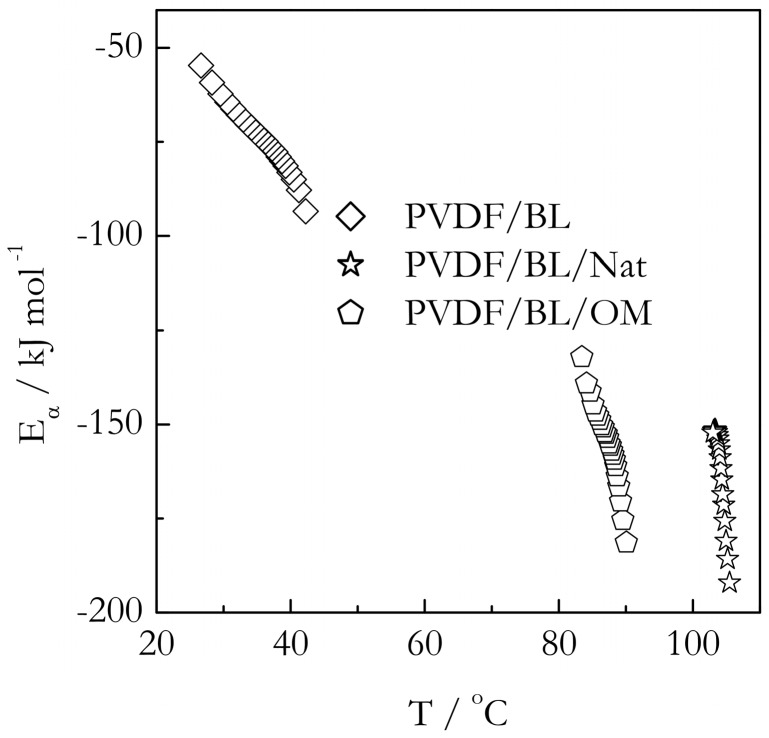
Temperature dependence of activation energy for gelation of PVDF/BL in bulk (diamonds) and confined systems PVDF/BL/Nat (stars) and PVDF/BL/OM (pentagons). Confinement is to 9 nm pores.

Figure 9 and Figure 10 demonstrate the temperature dependencies of the isoconversional activation energy for gelation in the bulk and nanoconfined systems. As demonstrated earlier, the effect of the pores’ size on the gelation temperature is very minor. Therefore, the nanoconfined system with the pore diameter of 9 nm has been selected as a representative example. The first thing immediately noticeable in Figure 9 and Figure 10 is that the activation energies are negative. Negative values of the isoconversional activation energy have been reported before for gelation of aqueous gelatin solutions [26]. They are also commonly found in the crystallization of polymer melts [55] as well as in the crystallization of salt solutions [56]. Since, by its meaning, *E_α_* is an effective value, its negative quantity is a simple reflection of the fact that the overall rate of the process increases with decreasing temperature. The existence of the negative *E_α_* follows directly from the Fisher-Turnbull model and Equation (2) derived from it. At small supercoolings (Δ*T*→0), the bracketed term in Equation (2) tends to infinity so that the difference in the right hand is necessarily negative. However, as supercooling increases, i.e., temperature lowers, the bracketed term decreases so that the difference, and, thus, the value of *E_α_*, increases, i.e., becomes less negative. This is exactly the type of dependencies we observe for all our systems. For each of the six systems presented in Figure 9 and Figure 10, *E_α_* increases with decreasing temperatures.

**Figure 11:**
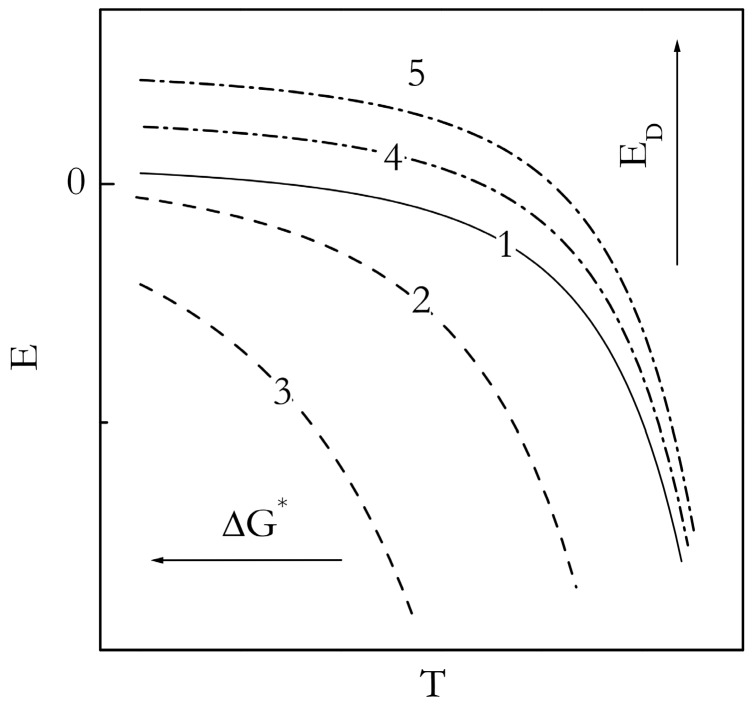
Schematic representation of the effect of the parameters of the Turnbull-Fisher equation (Equation (1)) on the temperature dependence of the activation energy (Equation (2)). Arrows show the direction of increase in ΔG* and *E_D_*. Curves 2 and 3 have the same *E_D_* as 1, but consecutively increasing values of ΔG*. Curves 4 and 5 have the same ΔG^*^ as 1, but consecutively increasing values of *E_D_*.

As stated earlier, an experimental *E_α_* vs. *T* dependence can possibly be fitted to Equation (2) to allow for estimating the parameters of the Turnbull-Fisher model (Equation (1)). Such fits have been successfully accomplished in analysis of the morphological solid-solid transitions [18,51], and coil-to-globule transition [52]. Nonetheless, in the present case of gelation, a meaningful fit does not appear possible. Although the experimental *E_α_* vs. *T* dependencies reflect correctly the general trend predicted by the Turnbull-Fisher model, their shapes deviate from the model ones. The model *E_α_* vs. *T* dependencies are depicted in Figure 11. It is seen that they have a convex upward shape. The experimental dependencies are either nearly linear or even have a convex downward shape. Apparently, the Turnbull-Fisher model is only a crude representation of the gelation process even though the latter carries so much similarity with crystallization. 

However, considering that the model is obviously capable of predicting the correct trends for the *E_α_* vs. *T* dependencies, it may still be used to obtain some semi-quantitative insights. As shown in Figure 11, the parameters of the Turnbull-Fisher model affect the position of the *E_α_* vs. *T* dependence in a different manner. An increase in the value of *E_D_* shifts the dependence to higher values of *E_α_*, whereas an increase in ΔG* shifts it to lower temperatures. Keeping this in mind, we can explain the trends observed experimentally (Figure 9 and Figure 10). For gelation of both TG and BL bulk systems, we observe that the *E_α_* values are larger, and the *E_α_* vs. *T* dependencies are shifted to lower temperatures relative to the nanoconfined counterparts. It means that for the nanoconfined systems, the occurrence of gelation at higher temperatures is associated with a lowering of the energy barrier to nucleation, ΔG*, which naturally causes acceleration of the process. Such lowering is typical in the case when the mechanism of nucleation changes from homogenous to heterogeneous [46,47]. This result is consistent with our idea that the nanoconfining surface causes the nucleation effect in the process of gelation. 

An unexpected result is that the lowering of the *E_α_* values for nanoconfined systems relative to the bulk ones appears to indicate that nanoconfinement also lowers the activation energy of diffusion. It is unexpected because under nanoconfined conditions, the polymer chains are squeezed into the space smaller than the diameter of an unperturbed coil and partially anchored by the interaction with the surface. This makes the polymer chain motion more hindered as though it faces a larger energy barrier. Apparently, the unexpected lowering of the activation energy of diffusion hints at a change in the diffusion mechanism. It should be kept in mind that the magnitude of the activation energy of diffusion is known [57,58] to decrease with decreasing the size of a diffusing molecule. That is, the short-range motion of small polymer chain segments would face much smaller energy barriers than the motion of the whole chain. Since the latter type of motion is virtually impossible under nanoconfined conditions, it is reasonable to assume that inside, the nanopores’ crosslinking of the polymers chains occurs at the expense of the short range segmental motion that has smaller activation energy. 

## 3. Materials and Methods

Poly(vinylidene fluoride) (PVDF) of average molecular weight 530,000 g mol^−1^ was purchased from Scientific Polymer Products Inc. (Ontario, NY, USA). 98% pure tetraethylene glycol dimethyl ether, also known as tetraglyme (TG), and >99% pure γ-butyrolactone (BL) were purchased from Alfa Aesar (Ward Hill, MA, USA) and Sigma-Aldrich (St. Louis, MO, USA), respectively. Ultrapure powdered silica gel (SiliaFlash) with an average particle size of ~50 µm and nominal pore diameters of 4, 6, 9, 15, and 30 nm was acquired from Silicycle, Inc (Quebec, QC, Canada). The Brunauer-Emmett-Teller (BET) analysis data for these samples have been provided by the manufacturer and presented in Table 1. Extensive microscopic studies [59] suggest that silica gel has the structure of interconnected pores of an approximately cylindrical shape. Organic modification of the silica samples was performed following the procedure of Anwander et al. [60]. The procedure involves converting the surface silanol groups of the native silica into trimethylsiloxane via a reaction with hexamethyldisilazane.

PVDF was dissolved in TG and BL to make 30 wt% solutions. The respective amounts of the polymer and solvent were weighed and placed in vials. The mixtures were stirred overnight with a magnetic stirrer at 90 °C for TG and 30 °C for BL. To ensure complete dissolution, the obtained solutions were sonicated for 3 h in a sonicating bath. The bulk gel was prepared by heating the solutions at 150 °C for five minutes and cooling them to room temperature. 

Nanoconfined gels were prepared in similar manner. After sonication, the PVDF solutions were introduced into the silica pores. The solution was taken in the amount necessary to fill all available volumes of silica samples. The pore volume per gram of silica was reported by the manufacturer. The mass of the solution needed to fill this volume was estimated via the density of the solutions. The respective mass of the solution was placed in a vial containing 50 mg of silica and stirred with a spatula. The solution quickly disappeared into the silica pores so that the obtained sample had the appearance of dry powder. Note that the polymer chains are capable of readily entering the pores whose size is markedly lower than the gyration diameter (2R_g_) because they are pulled into the pores by strong capillary action. The latter, in combination with high chain flexibility and the ability of chains to reptate (to engage in the snake-like motion) [61], enables the polymer chains to enter and move inside very narrow channels. For PVDF used in this study, the R_g_ value should be around 34 nm as estimated by linear interpolation of the R_g_ values measured in dimethyl sulfoxide for PVDF of molecular weights of 322,000 and 700,000 g mol^−1^ [62]. The polymer solutions inside silica were turned into the gels by heating at 150 °C for five minutes and cooling to room temperature.

The bulk and nanoconfined gels of PVDF were monitored using heat flux DSC (Mettler-Toledo, Greifensee, Switzerland). Indium and zinc standards were used to perform temperature, heat flow, and tau-lag calibrations. Melting of deionized water was used for calibration adjustment. The experiments were performed in the atmosphere of nitrogen flow (80 mL min^−1^). The samples were studied in closed 100 µL Al crucibles. The sample masses of the bulk gel were ~25 mg. Nanoconfined gel samples weighed approximately 50–60 mg and contained around 30 mg of the gel. The temperature program for the DSC was set as follows. To erase thermal history, samples were heated at 10 °C min^−1^ to 180 °C and held isothermally for 10 min to secure complete gel melting. Next, the samples were cooled to 25 °C (TG) or to 1 °C (BL) at 0.5, 1, 2, 4, and 8 °C min^−1^_._ Although the sample masses were relatively big, the maximum deviation of the sample temperature from the reference temperature was always less than 0.5 °C. All DSC measurements were done in triplicates.

The gel samples were also characterized by infrared and X-ray techniques. Infrared spectra were recorded using a Bruker Alpha II FTIR spectrometer (Billerica, MA, USA) operating in the transmission mode. Seventy scans at a resolution of 4 cm^−1^ were averaged to obtain each spectrum. XRD results were obtained using a Malvern Panalytical Empyrean X-ray diffractometer (Westborough, MA, USA) using a Bragg Brentano High Definition (BBHD) optics with a wavelength 1.54184 Å operated at 45 kV. The samples were scanned in the 2θ range of 10° to 45° with a step interval of 0.0131°.

## 4. Conclusions

The study has been driven by the hypothesis that gelation of polymer solutions confined to nanopores can be affected by the size and surface effects. Constricting the polymer chain to nanodimensions should decrease its mobility and, thus, hinder gelation. The presence of the nanoconfining surface should initiate heterogeneous nucleation and promote gelation. Calorimetric measurements on PVDF systems have revealed that both effects take place. Gelation of nanoconfined systems is accelerated as seen from a significant increase in the gelation temperature relative to that seen in the bulk systems. The effect is also found to be stronger in the native pores than in the organically modified ones, i.e., in the situation when the polymer-surface interactions are stronger. On the other hand, the hindrance of gelation is revealed in the significantly smaller heat of gelation measured for nanoconfined systems than in the bulk ones. The obtained results suggest that gelation is accelerated significantly in the vicinity of the pore surface, but retarded dramatically in the middle of the pore volume. Kinetic analysis of the data indicates that nanoconfined gelation faces lower energy barriers to both nucleation and diffusion. This is consistent with changes in the gelation mechanism from homogeneous to heterogeneous nucleation as well as from long to short segment mobility of the polymer chains.

## Figures and Tables

**Table 1:** Basic parameters of the porous silica.

Measured Values			
Nominal Pore Diameter (nm)	Pore Diameter (nm)	Surface Area (m^2^ g^−1^)	Pore Volume (cm^3^ g^−1^)
4	3.9	598	0.60
6	5.7	496	0.71
9	10.0	358	0.80
15	16.7	285	1.19
30	28.4	175	1.24
